# Kyasanur Forest disease virus non-mouse animal models: a pilot study

**DOI:** 10.1186/s13104-020-05137-8

**Published:** 2020-06-15

**Authors:** A. M. Nikiforuk, K. Tierny, T. A. Cutts, D. K. Kobasa, S. S. Theriault, B. W. M. Cook

**Affiliations:** 1 Applied Biosafety Research Program, National Microbiology Laboratory, Public Health Agency of Canada, 1015 Arlington Street, Winnipeg, MB R3E 3R2 Canada; 2 Special Pathogens Program, National Microbiology Laboratory, Public Health Agency of Canada, 1015 Arlington Street, Winnipeg, MB R3E 3R2 Canada; 3 Department of Medical Microbiology, and Infectious Diseases, The University of Manitoba, 745 Bannatyne Avenue, Winnipeg, MB R3E 0J9 Canada; 4 Department of Microbiology, The University of Manitoba, 213 Buller Building, Winnipeg, MB R3T 2N2 Canada; 5 Present Address: Cytophage Technologies Inc, 26 Henlow Bay, Winnipeg, MB R3Y 1G4 Canada

**Keywords:** Kyasanur Forest disease virus, Tick-borne, Animal model, Flavivirus, Guinea pig, Hamster, Ferret

## Abstract

**Objectives:**

Mouse models have delivered variable recapitulation of Kyasanur Forest disease (KFD) pathology and consistently demonstrated neurological involvement which may be a limited feature of human disease. With the purpose of more accurately modelling human disease progression we infected several small-mammalian models: guinea pigs, hamsters and ferrets with a titered infectious dose of Kyasanur Forest disease virus (KFDV). Clinical indicators of disease severity were observed for seventeen days, on day eighteen a visual post-mortem analysis of visceral organs was conducted. Viral load in selected tissues was measured to infer disease signs and the establishment of viral replication.

**Data description:**

Daily monitoring did not reveal any observable signs of illness; weight loss was minimal across species and gross pathology did not indicate severe viral infection. Tissue specific tropism and establishment of viral infection was monitored by quantitative real-time polymerase chain reaction (qRT-PCR). No viral replication was detected in ferrets (n = 0/3), but was present in the spleen of guinea pigs (n = 3/3) and the brain of hamsters (n = 3/3). Low levels of viral RNA were detected in multiple hamster tissues (kidney, liver, lung and spleen) suggesting the possibility of viral tropism and possible adaptation to the host. No serological tests were performed.

## Objective

In humans, Kyasanur Forest disease (KFD) is described as a biphasic illness that presents with flu-like symptoms in the first stage and may advance to a second stage with hemorrhagic fever manifestations and/or neurological abnormalities [1, 2]. Epidemiological studies and case reports have not provided clear evidence to ascertain if neurological symptoms are a common or rare sequalae in recovered individuals [3–6]. Attempts to establish viral replication in a mouse model have shown the absence of fever and progression of neurologic disturbance with viral RNA [7], viral antigen [8] and infectious virus [9] being detected in the brain. The high prevalence of neurological infection in mice may contradict human symptoms of disease progression. Thus, potentially making such models poor indicators of pathology, viral tropism and of diminished use in screening prophylaxis or vaccine candidates. This pilot study investigates the establishment of viral infection and etiologic pathology in non-mouse models (guinea pig (*Cavia porcellus*), hamster (*Mesocricetus auratus*) and ferrets (*Mustela putorius furo*)). Animals challenged with an infectious dose of Kyasanur Forest disease virus (KFDV) were monitored daily for visual signs of disease, febrility (ferret only), lethality, and weight loss. Post-mortem examination was performed to collect tissue from the: brain, kidney, liver, lung, spleen of each animal. Viral RNA was extracted from tissue samples and quantified by real-time RT-PCR. Presence of viral RNA was interpreted as evidence of viral tropism and replication. We hypothesized that one of the selected non-mouse models may act as a better model for human disease of KFDV infection than current mouse models.

## Data description

### Virus preparation and animal infection

KFDV isolate P9605 [10] was previously passaged nine times in suckling mouse brains, and two times within the Containment Level 4 (CL-4) Laboratory (Canadian Science Centre for Human and Animal Health, Winnipeg Canada) in Vero E6 cells. Viral supernatant was harvested from the second Canadian passage to obtain stocks for challenge of the non-mouse models.

Each animal species was housed separately in groups of three per cage: female (28–70 days old) outbred LVG Golden Syrian hamsters (Charles River Laboratories, New York, USA), female (26–58 weeks old) outbred Hartley guinea pigs (Charles River Laboratories, New York, USA) and female (650–800 g) specific pathogen-free (SPF) ferrets (Marshall BioResources, New York, USA) with subcutaneous implanted IPTT-300 temperature and ID transponder devices (BioMedic Data Systems Inc., USA). The procedures described herein, were approved by the Canadian Science Centre for Human and Animal Health-Animal Care Committee (animal use document H-14-010) adhering to the Canadian Council on Animal Care guidelines. Animals were fed and monitored daily for a 7-day acclimation period and for the duration of the experiment. Pre-challenge, each animal was anesthetized with 5% isoflurane in oxygen and 0.1 mL with 6.3 × 10^4^ TCID_50_ units of passage-two KFDV was intra-peritoneal-injected. The challenge dose was back titrated on Vero E6 cells. Observations of visual disease, weight loss and lethality were conducted for 16 days (hamsters and guinea pigs) or 17 days (ferrets). For ferrets, temperature was also recorded via the subcutaneous IPTT-300 sensors.

At the end of the observation period, animals were anesthetized with 5% isoflurane in oxygen and blood was collected. While under anesthesia, animals were euthanized (intra-cardiac injection of 120 mg/kg of pentobarbital) for gross pathology and tissue collection. Isolated tissues (brain, kidney, liver, lung and spleen) were inactivated with RLT and viral RNA was extracted with the RNeasy Plus Mini kit (Qiagen, Mississauga, Canada). Viral RNA was detected via quantitative real-time polymerase chain reaction (qRT-PCR) using the Roche LightCycler ^®^ 480 RNA Master Hydrolysis Probes and a previously published primer design [11, 12].

### Kyasanur Forest disease animal modelling

Throughout the course of observation, clinical signs of disease (ruffled fur, hunched posture, lethargy or loss of appetite) were not observed and all animals gained weight [14]. Temperature measurements from the ferrets fluctuated between 37.7 and 40.3 °C [14]. Upon post-mortem analysis, there were no apparent lesions or significant differences in organ weight. Assessment of viral RNA in isolated tissue samples (brain, kidney, liver, lung and spleen) revealed an absence of infection in ferrets (below limit of detection) and could only be found in the spleens of guinea pigs (Ct = 30.33–30.87) [13]. In contrast, viral RNA was detected across all sampled hamster tissues, with the exception of a single kidney sample. Viral RNA was detected in hamster brain tissue (Ct = 31.7–32.7) but not that of guinea pigs or ferrets (Ct => 40) [13] (Table 1). All data analysis was done in R version 3.6.3 [15].

**Table 1:** Overview of data files/data sets

Label	Name of data file/data set	File types (file extension)	Data repository and identifier (DOI or accession number)
Data File 1 [12]	KFD_CT.csv	.csv	figshare (10.6084/m9.figshare.12115797)
Data File 2 [13]	KFD_GE.csv	.csv	figshare (10.6084/m9.figshare.12115803)
Data File 3 [14]	KFD_KM.csv	.csv	figshare (10.6084/m9.figshare.12115812)
Data File 4 [15]	KFD_R_Rmd	.Rmd	figshare (10.6084/m9.figshare.12115818)

## Limitations

This pilot study contains several limitations including: small sample size, no replication for statistical inference, single dose-inoculation, and the presence of infectious virus cannot be directly inferred from the detection of nucleic acid [16]. Additionally, the P9605 isolate that was disseminated from India to many CL-4 laboratories in North America may be mouse-adapted [9] and is not a low passage isolate. From these findings we suggest that future attempts to model human-like disease from KFDV infection should consider: hamsters or immuno-compromised hamsters, a low passage wild-type isolate of KFDV or one rescued from an infectious clone system, should be inoculated for dose response, supernatants from tissue extraction should be passaged in a naïve animal to select for viral adaptation to the host. This pilot study demonstrates that hamsters could be preferred over guinea pigs or ferrets as a potential small non-mouse model of KFD pathology. It is unknown whether infected hamsters will recapitulate single phase or biphasic disease course and pathology.

## Data Availability

The data described in this Data note can be freely and openly accessed on figshare (https://figshare.com/) under: 10.6084/m9.figshare.12115797 [12], 10.6084/m9.figshare.12115803 [13], 10.6084/m9.figshare.12115812 [14], 10.6084/m9.figshare.12115818 [15].

## Abbreviations

KFD: Kyasanur Forest disease
KFDV: Kyasanur Forest disease virus
P9605: Passaged human isolate of Kyasanur Forest disease virus
CL-4: Containment level 4
SPF: Specific pathogen-free
RNA: Ribonucleic acid
qRT-PCR: Quantitative real-time polymerase chain reaction
IPTT: Implantable programmable temperature transponder
TCID_50_: Median tissue culture infectious dose assay
